# Spaces in the Brain: From Neurons to Meanings

**DOI:** 10.3389/fpsyg.2016.01820

**Published:** 2016-11-22

**Authors:** Christian Balkenius, Peter Gärdenfors

**Affiliations:** Cognitive Science, Lund UniversityLund, Sweden

**Keywords:** chorus transform, conceptual spaces, eye–hand coordination, population coding, radial basis function, similarity, stimulus generalization

## Abstract

Spaces in the brain can refer either to psychological spaces, which are derived from similarity judgments, or to neurocognitive spaces, which are based on the activities of neural structures. We want to show how psychological spaces naturally emerge from the underlying neural spaces by dimension reductions that preserve similarity structures and the relevant categorizations. Some neuronal representational formats that may generate the psychological spaces are presented, compared, and discussed in relation to the mathematical principles of monotonicity, continuity, and convexity. In particular, we discuss the spatial structures involved in the connections between perception and action, for example eye–hand coordination, and argue that spatial organization of information makes such mappings more efficient.

## 1. Introduction

Within psychology there is considerable evidence that many aspects of human perception and categorization can be modeled by assuming an underlying spatial structure (Shepard, 1987; Gärdenfors, 2000). A paradigmatic example is the color space (Vos, 2006; Renoult et al., 2015), but also, for example, the emotion space (Russell, 1980; Mehrabian, 1996) and musical space (Longuet-Higgins, 1976; Shepard, 1982; Large, 2010) have been extensively studied. Within cognitive linguistics, such spaces are also assumed to be carriers of meaning. For example, Gärdenfors (2000, 2014) has proposed that the semantic structures underlying major word classes such as nouns, adjectives, verbs and prepositions can be analyzed in terms of “conceptual spaces.”

For some of the psychological spaces, there exist models that connect neural structures to perception. For example, it is rather well understood how the different types of cones and rods in the human retina result in the psychological color space (see Renoult et al., 2015 for a review). The mammalian brain sometimes represents space in topographic structures. A clear example is the three layers in the superior colliculus for visual, auditory and tactile sensory inputs (Stein and Meredith, 1993). Another example of a topographic representation is the mapping from pitch to position in the cochlea and the tonotopic maps of auditory cortex (Morel et al., 1993; Bendor and Wang, 2005).

For most psychological spaces, however, the corresponding neural representations are not known. Our aim in this article is to investigate the hypothesis that also other representing mechanisms in the brain can be modeled in terms of spatial structures, even if they are not directly mapped onto topographic maps. We present some neuronal representational formats that may generate the psychological spaces. We want to show how psychological spaces naturally emerge from the underlying neural spaces by dimension reduction that preserve similarity structures and thereby preserve relevant categorizations. In this sense, the psychological and the neural spaces correspond to two different levels of representation.

Furthermore, we argue that spatial representations are fundamental to perception since they naturally support similarity judgments. In a spatial representation, two stimuli are similar to each other if they are close in the space (Hutchinson and Lockhead, 1977; Gärdenfors, 2000). Spatial representations also help generalization since a novel stimulus will be represented close to other similar stimuli in the space, and will thus be likely to belong to the same category or afford the same actions.

One of the main tasks of the brain is to mediate between perception and action (Churchland, 1986; Jeannerod, 1988; Stein and Meredith, 1993; Milner and Goodale, 1995). We argue that this task is supported by spatial representations. When both the sensory input and the motor output use a spatial representation, the task of mapping from perception to action becomes one of mapping between two spaces. To be efficient, spatial representations need to obey some general qualitative constraints on such a mapping. We focus on continuity, monotonicity, and convexity.

In the following section we present some basic psychological spaces and possible connections with neural representations. In Section 3, the role of similarity in psychological spaces, in particular in relation to categorization is presented and conceptual spaces are introduced as modeling tools. Section 4 is devoted to arguing that spatial coding is implicit in neural representations, in particular in population coding. In Section 5, we show how spatial structures are used in mappings between perception and action. Some computational mechanisms, in particular the chorus transform, are discussed in Section 6.

## 2. Basic psychological spaces

We share many psychological spaces with other animals. In this section, we briefly present some of the most basic spaces and outline the representational formats. First and foremost, most animal species have some representations of the external physical space. Even in insects such as bees and ants, one can find advanced systems for navigation (Gallistel, 1990; Shettleworth, 2009). However, the neuro-computational mechanisms that are used vary considerably between species. Mammals have a spatial representation system based on place cells in the hippocampus that are tuned to specific locations in the environment such that the cell responds every time the animal is in a particular location (O'Keefe and Nadel, 1978). This system is complemented by the grid cells in the entorhinal cortex that show more regular firing patterns that are repeated at evenly spaced locations in the environment (Moser et al., 2008). Taken together, the responses of these cells represent a location in space. This code is redundant in the information theoretical sense since many more neurons are used than would be strictly necessary to represent a point in three-dimensional space. One reason for this is that a redundant coding is less sensitive to noise, but it also supports the spatial computations made by the brain as we will see in Section 4.

**Figure 1:**
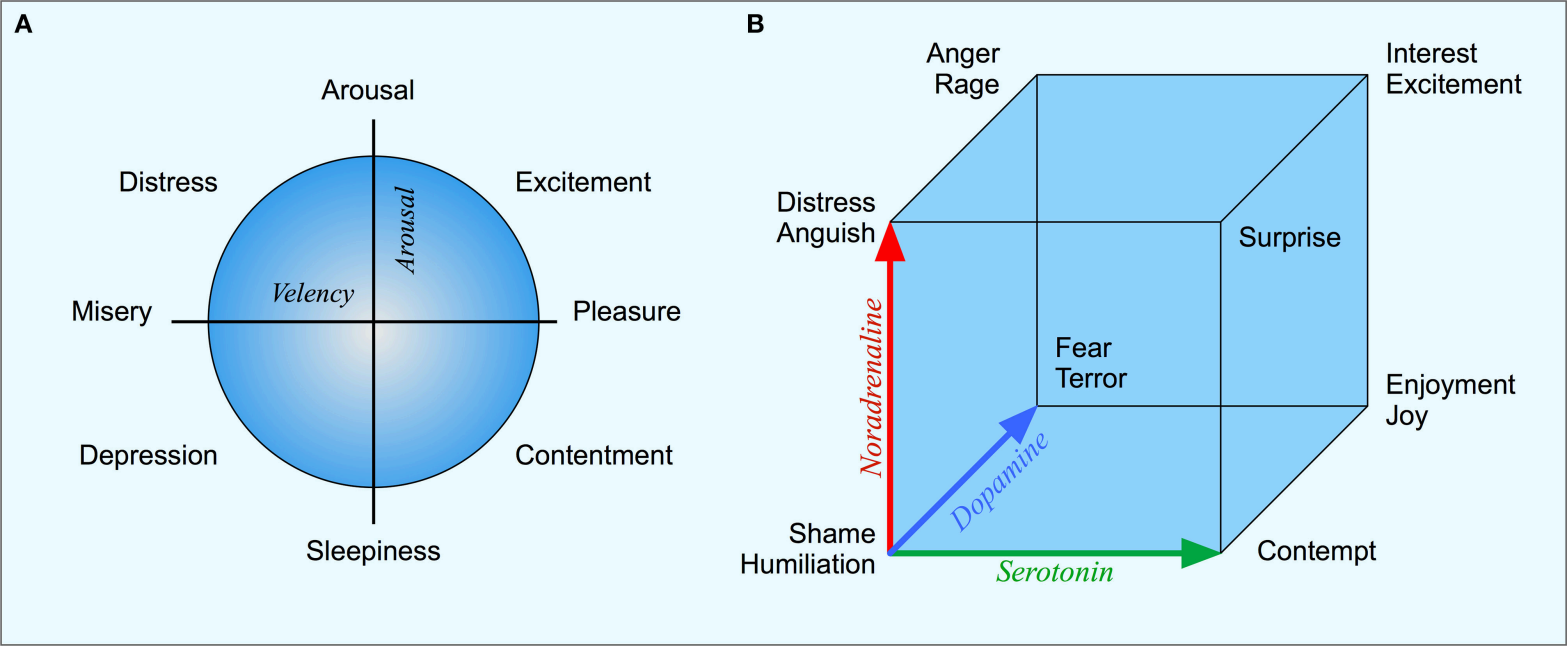
**(A)** Russell's circumplex with the two basic dimensions of valency and arousal and different emotions arranged in a circular structure. **(B)** Lövheim's emotion cube where the three axes represent the levels of dopamine, noradrenaline, and serotonin respectively.

A second example is the emotion space that is shared with many animal species. Mammals, birds, and other species show clear indications of at least fear, anger and pleasure and there are evolutionarily old brain structures that regulate these emotions and their expressions. For the psychological space of human emotions, there exist a number of models. Many of these models can be seen as extensions of Russell's (1980) two-dimensional circumplex (Figure 1A). Here, the emotions are organized along two orthogonal dimensions. The first dimension is valency, going from pleasure to displeasure; the second is the arousal-sleep dimension. Russell shows that the meaning of most emotions words can be mapped on a circumplex spanned by these two dimensions. Other models of psychological emotion space sometimes include a third dimension, for example a “dominance” dimension that expresses the controlling nature of the emotion (Mehrabian, 1996). For example while both fear and anger are unpleasant emotions, anger is a dominant emotion, while fear is non-dominant.

In relation to the topic of this paper, a central question concerns what are the neurophysiological correlates of the psychological emotion space. A recent hypothesis is the three-dimensional emotion cube based on neuromodulators proposed by Lövheim (2012), where the axes correspond to the level of serotonin, dopamine and noradrenaline respectively. By combining high or low values on each of the dimensions, eight basic emotions can be generated. For example, “fear” corresponds to high dopamine, low serotonin and noradrenaline, while “joy” corresponds to high noradrenaline, high serotonin and dopamine (see Figure 1B). The mapping between the representation in terms of neurotransmitters and the psychological emotion space remains to be empirically evaluated, but Lövheim's model presents an interesting connection between brain mechanism and the psychological emotion space. Unlike the coding of physical space, this representation has a direct relation between the underlying physiological variables, the transmitter substances, and the psychological emotion space.

A third example of a psychological space that is shared between many species is the color space. The human psychological color space can be described by three dimensions: The first dimension is hue, which is represented by the familiar color circle. The second dimension of color is saturation, which ranges from gray (zero color intensity) to increasingly greater intensities. The third dimension is brightness, which varies from white to black and is thus a linear dimension with end points. There are several models of this human psychological space that differ in some detail concerning the geometric structure, but they are all three dimensional (Vos, 2006).

In other animal species, the psychological color space has only been investigated, via discrimination tasks, for a limited number of species (Renoult et al., 2015). However, the dimensionality of the space varies from one-dimensional (black-white) two-dimensional (in most mammals), three-dimensional (e.g., in primates), to four-dimensional (in some birds and fish). For example, some birds with a four-dimensional space can distinguish between a pure green color and a mixture of blue and yellow, something that most humans cannot (Jordan and Mollon, 1993; Stoddard and Prum, 2008).

The next question then becomes how these various psychological color spaces can be grounded in the neurophysiology of the vision systems of different species. The retinas of tri-chromats such as humans have three types of cones that generate color perception: short wavelength (blue), medium wavelength (green) and long wavelength cones (red). Tetra-chromats typically have an additional type of cone that is sensitive to ultra-violet light (Endler and Mielke, 2005). Although every photoreceptor is tuned to a particular wavelength of light, its response intertwines its light intensity with spectral content (Hering, 1964). A change in photoreceptor response can be the results of a change in light intensity as well as a change in color. It is only when the responses of receptors with different tuning are combined that the brain can distinguish between brightness, saturation, and hue.

There exist different theories regarding the connection between the signals from the cones and the rods and the perceived color. One is the opponent-process theory that claims that for tri-chromats there are three opponent channels: red vs. green, blue vs. yellow, and black vs. white. The perceived color is then determined from the differences between the responses of the cones (Hering, 1964). The theory has received support also in several animal species with known tri-chromacy, for examples in primates, fish and bees (see Svaetichin, 1955; De Valois et al., 1958; Backhaus, 1993). For tetra-chromats, a similar theory has been proposed (Endler and Mielke, 2005; Stoddard and Prum, 2008).

It is interesting to note that even though both the receptor space and the psychological color space are both of low dimension, they are not the same. For example, the subjective experience of a color circle has no correspondence in sensory physiology. For humans, the color coded at the receptor level is a cube while the psychological space has the shape of a double cone. None of these spaces are a direct representation of the physical light spectrum.

## 3. Models of psychological spaces

### 3.1. Similarity as a central factor

Perhaps the most important cognitive function of the brain is to provide a mapping from perception to action (Milner and Goodale, 1995). In the case of simple reflex mechanism, the mapping is more or less fixed and automatic. In most cases, however, the mapping has to be learned (Schouenborg, 2004) and it is a function not only of the current perception, but also of memory and context (Bouton, 1993). It is central that such a mapping can be learnable in an efficient way. A general economic principle for cognition is that similar perceptions should lead to similar actions. Therefore, similarity should be a fundamental notion when modeling the mapping from perception to action.

In the behavioristic tradition, connections between stimuli and responses were investigated. This research lead to the principle of stimulus generalization that says that, after conditioning, when the subject is presented with a stimulus that is similar to the conditioned stimulus, it will evoke a similar response (Hanson, 1957, 1959). For example, work by Shepard (1957) was seminal in showing that stimulus generalization can be explained in terms of similarity between stimuli. Within this tradition, it was seldom studied what made a stimulus similar to another. What was meant by similarity was taken for granted or induced by varying a physical variable (Nosofsky and Zaki, 2002).

If we leave the behavioristic tradition and turn to more cognitively oriented models, a general assumption is that the connection between stimuli and responses is mediated by a categorization process and that it is the outcome of the categorization that determines the action to be taken. For example, stimuli are categorized as food or non-food, which then determines whether an act of eating will take place.

There exist several psychological category learning models based on similarity. Some are based on forming prototypes of categories (Rosch, 1975; Gärdenfors, 2000). One way of using prototypes to generate concepts is by Voronoi tessellations (see next subsection) that are calculated by placing any stimulus in the same category as the nearest prototype (Gärdenfors, 2000). Other category learning methods are based on learning a number of exemplars for the different concepts in a domain. (Nosofsky, 1988; Nosofsky and Zaki, 2002). Then a new stimulius is categorized as the same as its nearest neighbor among the exemplar. This is also a technique that is commonly used for pattern recognition in an engineering context (Cover and Hart, 1967).

A general problem for such categorization models is that only for special types of stimuli it is known how the underlying similarity structure can be described. For most stimuli, the modeling will have to be based on hypotheses. The idea that similar perceptions should lead to similar action can, however, be formulated in terms of some general principles that a mapping from perceptions to actions should fulfill. In mathematical terms, the principles can be described as monotonicity, continuity and convexity. Monotonicity means that an increase in a perceptual variable should correspond to an increase in an action variable. For example, if an object B is perceived as being further away than object A, then the agent must reach further to grasp B than to grasp A. Continuity means that small changes in a perceptual variable should correspond to an small change in an action variable. Again eye–hand coordination provides an example: When reaching for an object, the agent makes small adjustments to hand movements in order to adjust for small perceptual discrepancies between hand and object. Convexity means that closed regions of a perceptual space are mapped onto a closed region of action space. To continue with the reaching example, this requirement entails that if object C is located between objects A and B, then the motor signals to reach C should also lie between the motor signals to reach A and to reach B. Even if these three requirements do not determine the mapping from perceptions to actions, they provide strong constraints on such a mapping. The important thing to notice is that once perceptions and actions are spatially represented, a continuous mapping from perception to action typically also fulfills the criteria of monotonicity and convexity. These properties are also important from the perspective of control theory, for example when a robot needs to interpolate between learned movements in novel situations (Schaal and Atkeson, 2010).

Furthermore, when an agent is learning, for example, to coordinate the information from the eyes with the actions of the hands, the fact that the mapping satisfies these conditions potentially makes the learning procedure considerably more efficient. Even with little training, it would be possible to interpolate between already trained mappings from eye to hand and to test a movement that likely is close to the correct one. Under ideal conditions, it is sufficient to have learned how to reach three points on a plane to be able to reach any position on that plane. Other points can be reached by interpolating (or extrapolating) from the movements that reaches each of these three points. Although such interpolation does not necessarily lead to a perfect behavior, it is a good starting point and as more movements are tested the mapping will quickly converge on the correct one.

### 3.2. Conceptual spaces

A modeling problem is how psychological and neurological spaces can best be represented. Gärdenfors, 2000 proposes that categories can be modeled as convex regions of a conceptual space. A psychological conceptual space consists of a number of domains such as space, time, color, weight, size, and shape, where each domain is endowed with a particular topology or geometry. Convexity may seem a strong assumption, but it is a remarkably regular property of many perceptually grounded categories, for example, color, taste, and vowels. Although a main argument for convexity is that it facilitates the learnability of categories (Gärdenfors, 2000), it is also crucial for assuring the effectiveness of communication (Warglien and Gärdenfors, 2013). In this article, we focus on the role of convexity in mappings from perception spaces to action space.

**Figure 2:**
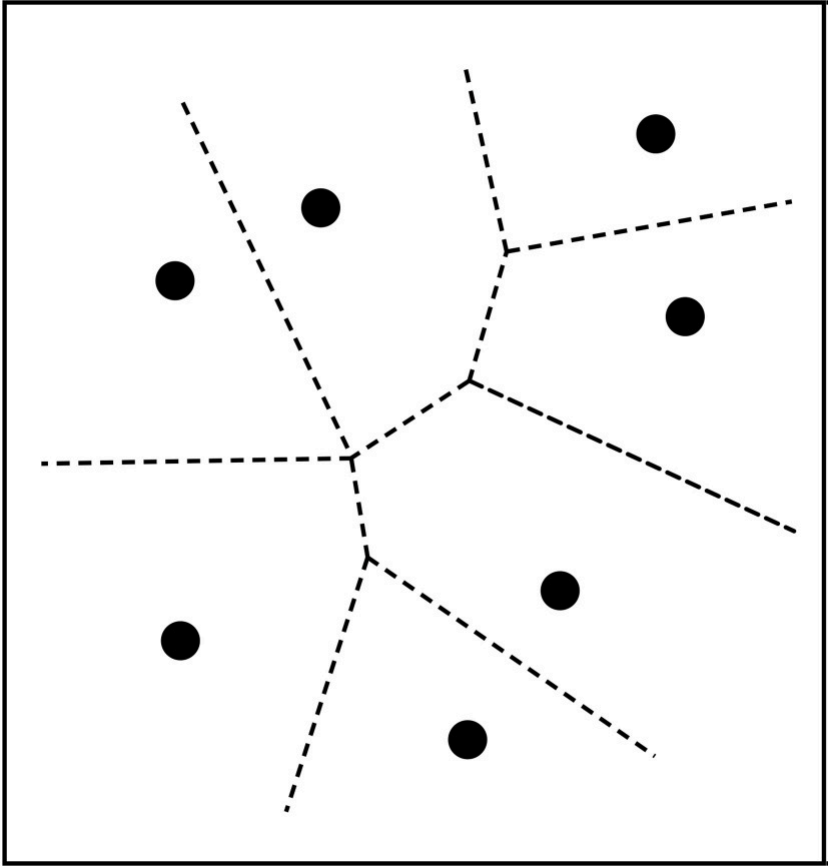
**Voronoi tessellation of the plane into convex categories**. Each point represents a prototype and the lines show the borders between the categories.

There are interesting comparisons to make between analyzing categories as convex regions and the prototype theory developed by Rosch and her collaborators (Rosch, 1975; Mervis and Rosch, 1981; Lakoff, 1987). When categories are defined as convex regions in a conceptual space, prototype effects are to be expected. Given a convex region, one can describe positions in that region as being more or less central. Conversely, if prototype theory is adopted, then the representation of categories as convex regions is to be expected. Assume that some conceptual space is given, for example, the color space; and that the intention is to decompose it into a number of categories, in this case, color categories. If one starts from a set of categories prototypes—say, the focal colors—then these prototypes should be the central points in the categories they represent. The information about prototypes can then be used to generate convex regions by stipulating that any point within the space belongs to the same categories as the *closest* prototype. This rule will generate a certain decomposition of the space: a so-called Voronoi tessellation (see Figure 2). The illustration of the tessellation is two-dimensional, but Voronoi tessellations can be extended to any arbitrary number of dimensions. An important feature of Voronoi tessellations is that they always generate a convex partitioning of the space.

The prototype structure of concepts is also central for modeling meanings of words. Gärdenfors (2000, 2014) develops a semantic theory where elements from the main word classes are mapped onto convex regions of domain or convex sets of vectors over a domain. This way of representing word meanings can explain many features of how children learn their first language. Again, the low-dimensional structure of the domains are essential for rapid learning of new word meanings (Gärdenfors, 2000).

## 4. Spatial coding is implicit in neural representations

We next turn to a more general account of how space may be neurally represented. We suggest that a spatial coding is implicit in most neural mechanisms, and that concepts of distances and betweenness are readily applicable to such codes.

As a first example, we look at the neurons in motor cortex. These neurons code for the direction of movement using a *population code* where each individual neuron is tuned to movement in a particular direction (Georgopoulos et al., 1988) and modulated by distance (Fu et al., 1993). In a population code, a stimulus or a motor command is coded by the joint activities of a set of neurons. Before the movement, the response of each cell is proportional to the angle between the direction vector represented by that cell and the direction of the following movement. Cells with vectors close to the movement direction will respond more than cells that code for different movement directions.

**Figure 3:**
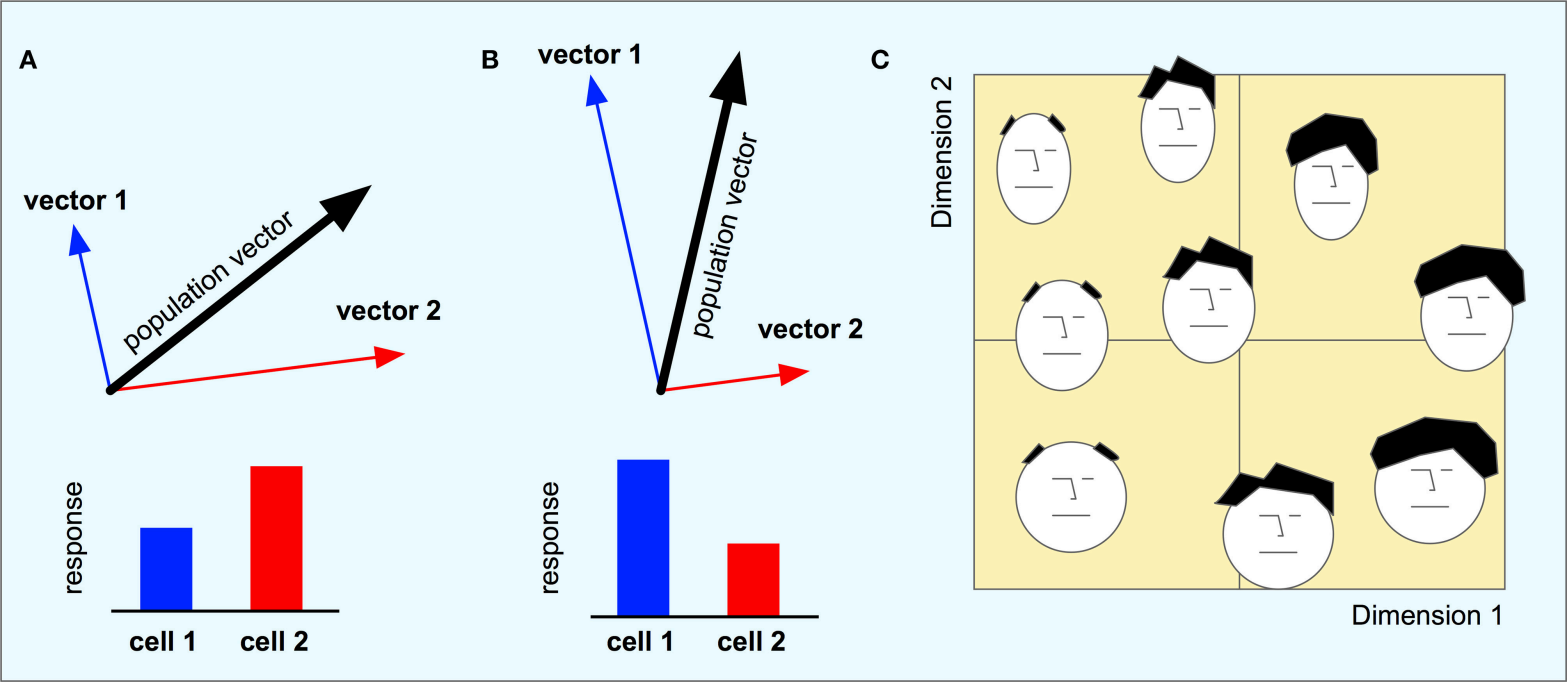
**(A,B)** The direction vectors from a minimal population of two cells are combined into a population vector. Each cell codes for a particular movement direction and the responses of the two cells weigh together the two vectors into a combined population vector that corresponds to the subsequent movement direction. When the response of cell 2 is higher than that of cell 1, the population vector will point in a direction that is closer to vector 2 than that of vector 1. When cell 1 has the higher response, the population vector will be more aligned with vector 1. **(C)** A hypothetical face space of the type found by Young and Yamane (1992). Different faces are arranged along two dimensions where faces that are similar to each other are located close to each other in the space.

The set of neurons can be seen as a basis for a highly redundant high-dimensional coding of a low-dimensional vector space for movement direction. The responses of all neurons taken together represent a population vector that can be computed by adding together the direction vectors of each individual neuron weighted by its response magnitude (Figures 3A,B). The population vector is thus the low-dimensional “decoding” of the high-dimensional population code.

The similarity between two population codes can be calculated by considering the population codes as vector in the high-dimensional space. The similarity is defined by the cosine of the angle between these vectors. This similarity measure varies between 0 and 1, where 1 indicates identical population codes, and a value of 0 indicates two maximally dissimilar codes. This is different from calculating the similarity between the population vectors that lie in the low-dimensional space. A fundamental aspect of the population coding is that population codes that are similar using this measure in the high-dimensional neural space will produce population vectors that are also similar in the low-dimensional movement space.

Population codes are not only used for motor coding but are also used for perceptual tasks. In their seminal study of population coding of human faces in the anterior inferotemporal cortex (AIT) and anterior superior temporal polysensory area (STP) of macaque monkeys, Young and Yamane (1992) showed that the recorded responses of these brain regions contain information about the identity (AIT) and possibly familiarity (STP) of the faces the monkey is viewing. The responses of a large number of neurons to different faces were recorded. Using multidimensional scaling, they mapped the recordings onto a lower dimensional space. The dimensions of this space are not visible when looking at a single neuron that responds preferably to a single stimulus and gradually decreases its response as the stimulus moves away from the preferred one. However, by looking at the low-dimensional code, they were able to show that two dimensions explained most of the variation in the population code for each of the two brain regions.

This implies that the macaque brain implicitly uses a low-dimensional space to code different faces. Although a high-dimensional population code is used, most of the information is contained in a small number of dimensions. Each face is coded in a unique location in this space, and faces coded close to each other in the space share visual characteristics such as the amount of hair and the general shape of the face. The distance between points in this low-dimensional space represents the similarity between the coded faces (Figure 3C) and may correspond to the psychological face space of the monkey.

For both examples of population coding described above, the underlying space appears to be two-dimensional, but this is clearly an artifact of the experimental details. In Georgopoulos' experiments, the monkey moved its arm in two dimensions, the vectors found are consequently also two-dimensional, but we must assume that the same principle holds for movement in three dimensions and possibly also for more complex movements that are extended in time and involves more degrees of freedom (Graziano et al., 2002). The only difference in this case is that a larger number of dimensions are necessary. Similarly, in the experiment by Young and Yamane, two dimensions were sufficient to capture most of the variation necessary to distinguish the different faces, but presumably, the monkey could have access to more dimensions had it been necessary to differentiate between the faces. The exact number of dimensions in neural representation is not important as long as a low-dimensional reduction of the space covers most of the information.

There are two ways to view the coding in the brain—one at a detailed level, the other at an aggregated level. The first is to look at each neuron individually. By systematically testing different stimuli, it is possible to find the stimulus that each neuron maximally responds to (Tanaka, 2003). In this case, the neuron is considered a detector tuned to that preferred stimulus. The preferred stimulus can be seen as the *prototype* for that neuron (Edelman and Shahbazi, 2012), and the more similar a stimulus is to that prototype, the stronger the neuron will react.

The other approach is to look at the whole population of neurons and view the activity pattern as a point in a high dimensional space. In this case, the response of each neuron is seen as a basis function and every stimulus is coded as a blend of these *basis functions*^1^. In this case, the responses of individual neurons are not necessarily meaningful on their own. Although these two views may look contrasting, they are actually two sides of the same coin and are both equally valid.

Although a population code consists of the activity of multiple neurons that are not necessarily located close to each other on the cortical surface, Erlhagen and Schöner (2002) have suggested that neurons that make up a stable activity pattern may be linked by mutual excitation in such a way that they functionally can be considered a point in a higher dimensional topographic space. This is a central component of the Dynamic Field Theory that studies the temporal dynamics of such activity patterns.

We have here looked at how low dimensional spaces are implicitly coded in a high dimensional population code, but the brain also constructs lower dimensional codes explicitly throughout the sensory system. This is often modeled as successive steps of dimensionality reduction, or compression, in hierarchical networks (e.g., Serre et al., 2007). In the semantic pointer architecture (Eliasmith, 2013), relatively low dimensional codes that are constructed in this way are used to define a “semantic space” where different concepts can be represented. The high dimensional representation at lower levels in the hierarchy can be partially reconstructed from the low-dimensional semantic pointer. Furthermore, the architecture allows for recursive binding through the operation of circular convolution. Unlike earlier methods using tensor operations (Smolensky, 1990), circular convolution does not increase the dimensionality of the representation and can be performed in several successive steps to produce deep embeddings (Blouw et al., 2015). Many other forms of binding mechanisms are discussed by van der Velde and De Kamps (2006). Common to all are that the individual constituents can have the spatial structure described above.

## 5. The use of spaces as mappings between perception and action

We next turn to neuro-cognitive models that include both the sensory and the motor side. Specifically, we want to show that sensory-motor mappings can be described as mappings between points in low-dimensional spaces, Here we only consider basic examples of sensory-motor mappings, but the principles we present are general.

A direct form of sensory to motor mapping is used when we keep our head stationary and let the eyes saccade to an object. The location of the object is captured in eye-centered coordinates and it is necessary to convert these into the appropriate motor commands to move the eyes to that location. This sensory-motor transformation can be seen as a mapping between two representational spaces, one for the object location and one for the movements of the eyes.

For a saccade, the mapping is relatively simple since every location on the retina could in principle be mapped to a unique motor command (Salinas and Abbott, 1995). When a target is detected on the retina, a motor command would be produced that would point the eye in the correct direction. There would thus be one eye direction vector for each retinal position. Here, the desired gaze direction is a function of the target location on the retina and the function satisfies the three conditions of monotonicity, continuity and convexity. As the target moves further away from the center of the eye, the required movement is larger and the mapping is thus monotone. A small change in the target position on the retina, requires a small change of the corresponding movement. The mapping is thus continuous. It also follows that the convexity criteria is met since the movements are mapped out in an orderly fashion over the retina. The movement to a target that is projected between two arbitrary points on the retina lies somewhere in-between the two movements required for the two points.

**Figure 4:**
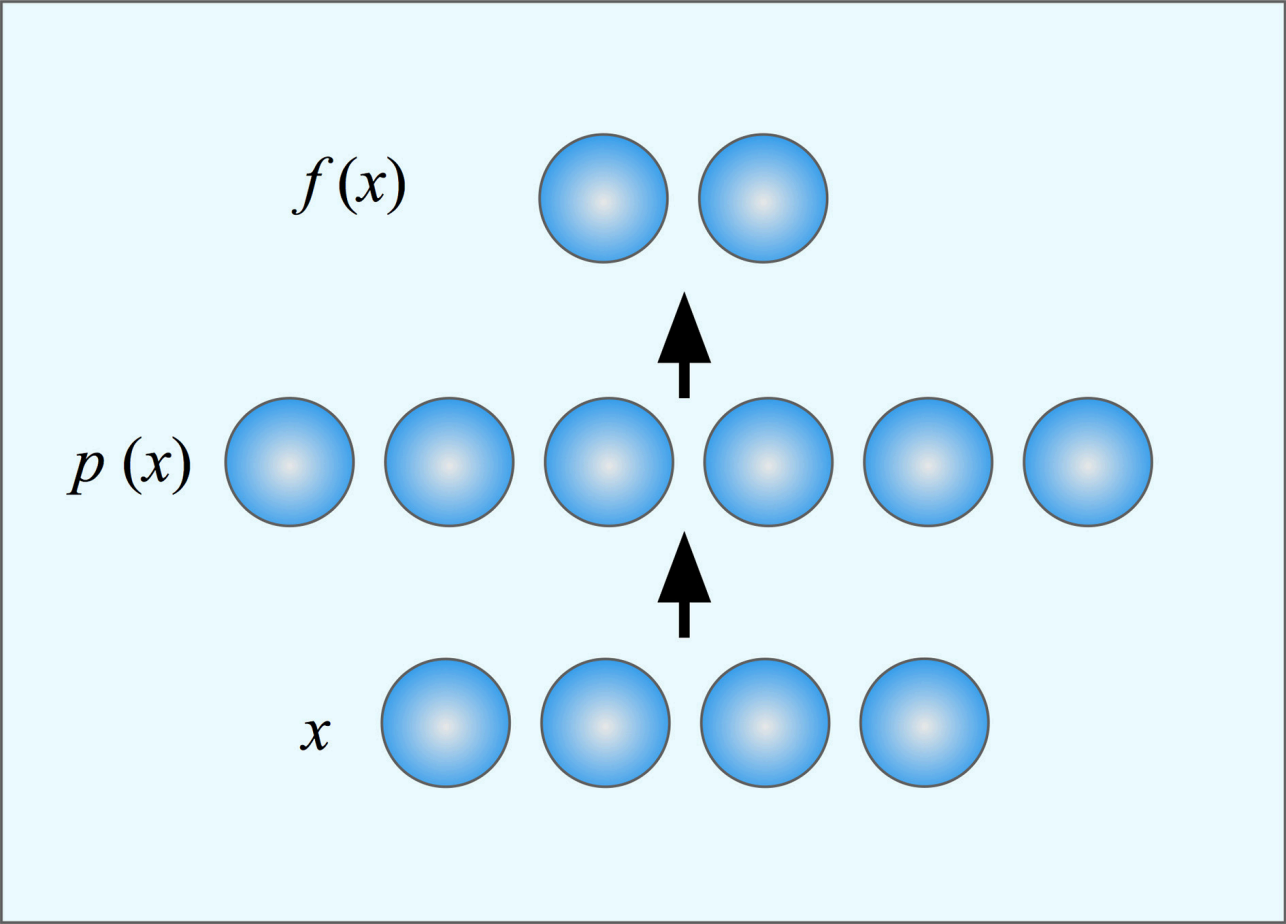
**A three-layer neural network**. The input x is mapped onto a population code *p*(*x*) in a hidden layer where each neuron (or basis unit) is tuned to different position in the input space (its prototype). The output function *f*(*x*) is computed by weighting together the responses of the units in the hidden layer. Different functions can be computed by weighting the outputs differently. Learning in the network consists of finding the appropriate weights for the desired function.

Deneve and Pouget (2003) suggested that mappings from sensory to motor systems could be performed using basis function maps. Such maps use basis functions to represent all possible stimuli in such a way that linear combinations of basis functions can compute any motor command. More specifically, they propose that the neurons of the supplementary eye field of the parietal cortex form a set of basis units. Each basis unit corresponds to a single prototype in the sensory space. The output from the unit codes the distance from the input to that prototype. The task for the subjects in their experiment was to saccade to the left or the right part of an object that appeared at an arbitrary location and orientation on the retina. This task is interesting since it requires that the whole object is identified before it is possible to localize its sides and thus it appears to require object-centered representations and as such a sequence of coordinate transformation would be necessary. However, Deneve and Pouget showed that this task can be performed as a single mapping by a three layer network where the middle layer consists of basis function units (Figure 4). The basis units work together so that inputs that match several basis units will produce an output that is a combination of the outputs from each of the individual basis units. A finite set of basis units can thus cover the whole input and output spaces. The responses of all these basis units together constitute a population code (Pouget and Snyder, 2000).

Since we know that the input can be described by a small number of variables (here the position and orientation of the object combined with the instruction to look at the left or right part of it), it is clear that the responses of the basis function units implicitly codes for this low-dimensional space. Similarly, the output is a point in a two dimensional space consisting of the possible targets for the saccade. We can thus interpret the operation of the network as a mapping from a four-dimensional to a two-dimensional space, although the computations are made implicitly in a high-dimensional space as a linear combination of basis unit responses.

Similar models have been proposed to explain the sensory-motor transformations necessary to reach for a visually identified object (Zipser and Andersen, 1988). For example, to point at a visual target the brain needs to take into account the position of the target on the retinas of the two eyes, the orientation of the head and eyes and the posture of the body. To compute the location of the target relative to the hand, the target must first be identified on the retina and then it is necessary to compensate for the location of the eyes relative to the hand and the rest of body. This can be viewed as a sequence of coordinate transformations, but it is also possible that, like in Deneve and Pouget's (2003) model, the target location could be found in a single step by mapping from a space coding retinal position and the positions of all the relevant joints. In either case, these computations can be made as mappings between population codes in different layers of a network (Zipser and Andersen, 1988; Eliasmith and Anderson, 2003).

The relative roles of retinal target position and joint angles can be seen in an experiment by Henriques et al. (2003). The experiment showed that reaching is easier when we look directly at the target compared to when the target is off-gaze. This indicates that the orientation of the eyes has a larger influence on the movement than the retinal position of the target and supports the idea that joint position are used in computations of spatial locations. The result is probably a consequence of the fact that we most often look directly at an object we try to reach.

In the brain, the mapping from the retinal position and eye direction to the external target location that controls reaching movements is believed to take place in the posterior parietal cortex (PPC) (Jeannerod, 1997). Zipser and Andersen (1988) looked at the responses of the neurons in area 7a of PPC and trained an artificial neural network on the mapping from eye direction and retinal position to head-centered coordinates. The network consisted of three layers where units in the first layer code for retinal position and eye direction. The activity of the output layer indicated the head-centered location of the target. The model produced similar response properties as the real neurons of PPC. The neurons in the hidden layer became tuned to retinal position but they are also modulated by eye position. Like the saccade control described above, this learned mapping fulfills the criteria of monotonicity, continuity and convexity.

The type of population coding that is found in area 7a, and that also emerges in the hidden layer of the model, is often called a *gain field* (Zipser and Andersen, 1988; Buneo and Andersen, 2006). Like other types of population coding, different neurons take care of different parts of the mapping and the final result is obtained by weighing together the contributions of each neuron (Figures 3, 4). However, a gain field is characterized by the fact that the neurons are primarily organized along only some of the input dimensions. For example, Zipser and Andersen (1988) found that neurons coding for target position were retinotopically tuned, but responded differently depending on the eye positions.

Salinas and Abbott (1995) also addressed the question of how the brain can transfer information from sensory to motor system using population codes. They investigated the coordinate transformations in visually guided reaching and proposed a model that uses a Hebbian learning mechanism to learn the sensory-motor mapping. Unlike Zipser and Andersen, they assume that the input space is already covered by a large set of prototypes coded by a set of basis units. They view the problem of eye–hand coordination as a form of function approximation where the problem is to find the appropriate weights for the outputs of each basis unit to obtain the desired mapping (Figure 4). They show how these weights can be learned using Hebbian learning and the general model they present can be used to describe arbitrary mappings between spaces.

We now turn to the slightly more complicated situation where reaching is followed by grasping an object. Here, we not only need to locate the target, we also need to shape the hand in the correct way both before reaching the object and subsequently to grasp it. Despite the added complexity, this too can be seen as a mapping between two spaces. In the case that only visual information is used, the input space codes for the location and the shape of the object while the output space minimally contains the movement direction for reaching, the parameters to preshape the hand and finally the force vectors to perform the grasping movement.

Although little is known about how shapes are represented in the brain, work in mathematics (Kendall, 1984) and computer graphics (Blanz and Vetter, 1999; Kilian et al., 2007) show that it is possible to design shape spaces where different shapes can be synthesized from combinations of basic shapes in a way that is similar to how basis units work together to represent a point in a space using a population code. For a known rigid object however, it is sufficient to code the orientation of the object. This can be done in a three dimensional space of the rotation angles that describes the orientation of the object^2^. The orientation can be represented in a way similar to position by a set of basis units that together code for all possible orientations of the object. Here the final mapping is between a six-dimensional space representing position and orientation to a space that represents the critical parameters of the reaching movement.

There are a number of spaces that could potentially be involved in eye–hand coordination. Depending on task constraints, the brain is thought to use both egocentric and allocentric representations of space (Crawford et al., 2004) and there is evidence that neurons in PPC code for targets in relation to both gaze (Batista et al., 1999) and hand (Buneo and Andersen, 2006). Investigating spatial representations for reaching in the superior parietal lobule (SPL) of PPC, Buneo and Andersen (2006) found evidence for representations of targets in both eye-centered coordinates and of the difference in position between the hand and target.

**Figure 5:**
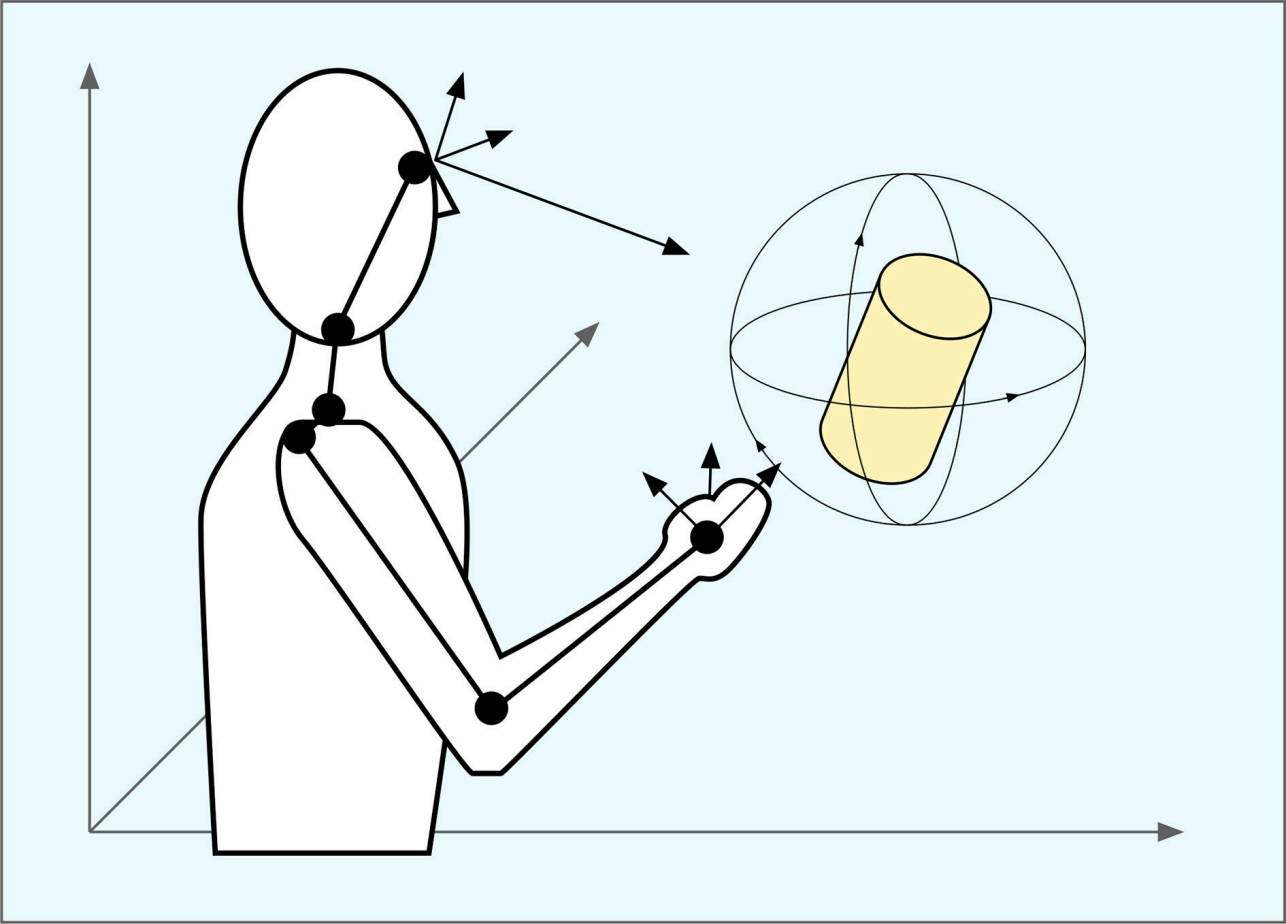
**Coordinate systems in eye–hand coordination**. The position of an object can be coded in in relation to allocentric space, the eye or the hand and potentially also other parts of the body. To reach and grasp the object, the brain must map representations of the object from a sensory space to a motor space in order to control the arm and hand in an appropriate way.

Figure 5 summarizes some of the coordinate systems involved in eye–hand coordination. The target object can be represented in either allocentric or one of the egocentric spaces. For reaching, an egocentric frame of reference is more suitable but as we have seen, there are several egocentric spaces corresponding at least to the eye and the hand, but it is likely that many more exist and presumably the brain is able to map freely between them. To grasp an object, its representation must be mapped on the space that contains the possible motor actions. The dimensionality of this space is high enough to contain all possible grasp movements, but still of limited dimensionality. For the brain to learn these mapping in an efficient way, it is necessary that where possible, these mappings fulfill the three conditions of monotonicity, continuity and convexity. We submit that population codes are used to make this possible.

## 6. Mechanisms

**Figure 6:**
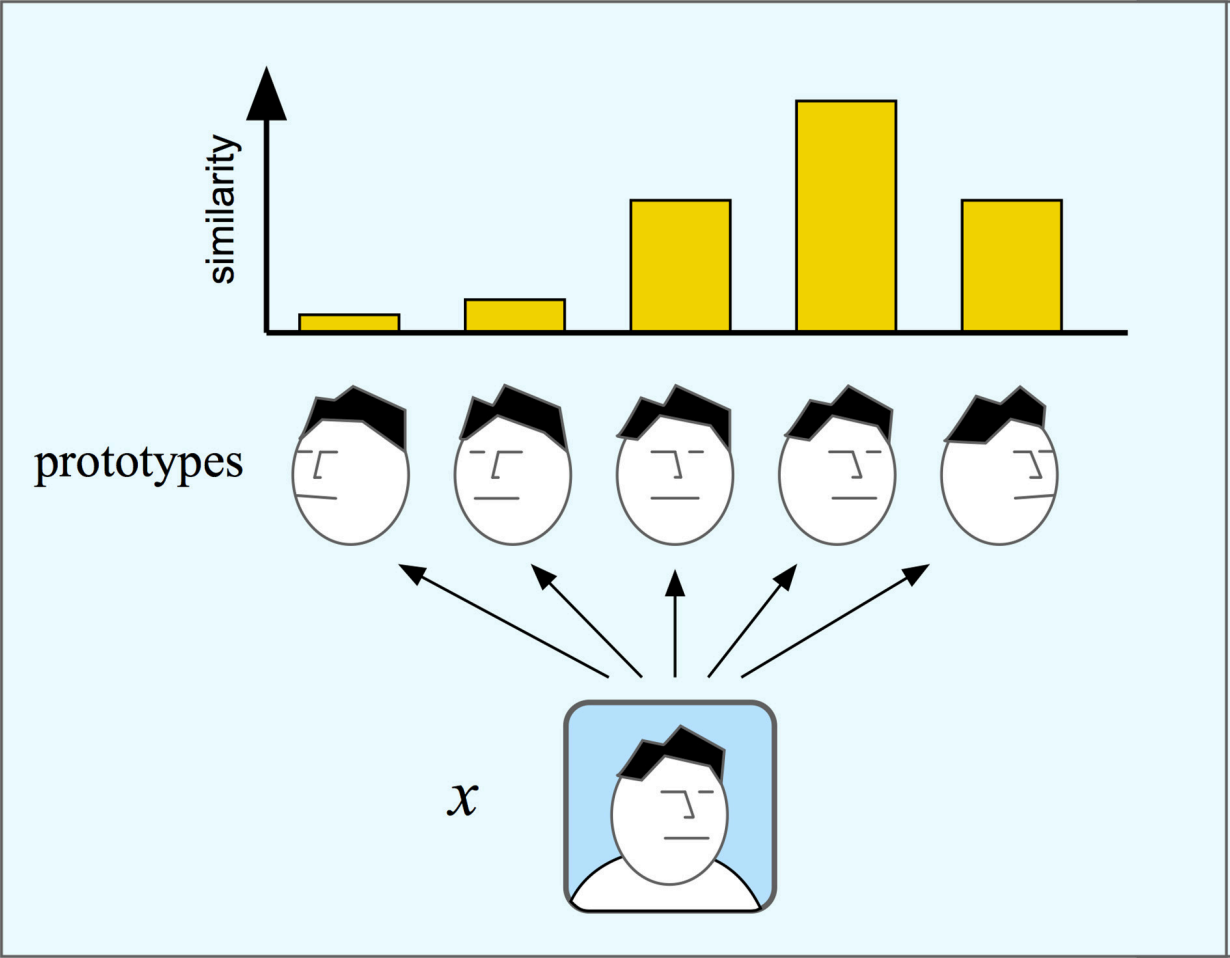
**In the chorus transform (and in RBF networks) an input is mapped to its similarity to a number or prototypes**. Here, an image x is compared to five face prototypes with different orientation. Each comparison produces one component of the chorus transform that will here consist of five similarity measures.

Population codes of spaces as described above are instances of a coding scheme where each input is coded by the distance to a number of prototypes. The optimal stimulus for each neuron, or basis unit, in the population can be considered the prototype for that unit. One such form of population coding is given by the *chorus transform* proposed by Edelman (1999) who calls it a “chorus of prototypes.” In the simplest case, the response of each unit is the similarity to the prototype measured by some suitable metric. Figure 6 shows an example of a chorus transform. The input to the transform is an image of a face. This face is compared to each of five face prototypes and the resulting transform is the set of similarity measurements. The chorus transform thus describes a type of population coding of the input.

An important property of the chorus transform is that it preserves Voronoi tessellations of the input space (Edelman, 1999, p. 268). It also approximately preserves the inter-point distances in the original space. This means that category boundaries in the input space are mostly preserved in the output space (Edelman and Shahbazi, 2012). This has several critical consequences for both neural coding and mappings of spatial representations:

*Stimulus generalization*. The spatial representation naturally supports *generalization* since novel stimuli will be coded by the similarity to known stimuli and the coding will gradually change if the stimulus gradually changes. There is thus a continuous mapping from stimulus properties to the representation of the stimulus.

*Discrimination*. Since discrimination borders are typically Voronoi borders and these are mostly preserved by the coding, it means that discrimination borders in the input space are preserved in the coding space.

*Categorization*. For the same reason, categories induced by the Voronoi tessellation are preserved in the population coding.

When looking at mapping between two spaces coded by a population of units we note that these properties of the chorus transform imply that a linear mapping from such a representation also have these properties. This has consequences for sensory-motor mapping between spaces:

*Interpolation*. Since similar stimuli are coded by similar population codes, similar stimuli will be mapped to similar motor outputs. This is equivalent to the continuity criteria introduced above.

*Sensory-motor categories*. When object categories are represented as Voronoi borders in the stimulus space, different stimulus categories can be mapped to different motor actions by a single mapping. When the input crosses the Voronoi border between two categories, so will the output, and in the same way a small part of the input space represents a particular category, a corresponding part of the output space can represent actions suitable for that category. Furthermore, since Voronoi borders are preserved in the mapping it follows that the convexity criteria is also fulfilled by these types of mappings.

The most commonly used computational architecture that uses the chorus transform is the *radial basis function* (RBF) network (Moody and Darken, 1989). This artificial neural network consists of three layers (Figure 4). The middle layer consists of units that are tuned to different stimulus prototypes and their response is maximal when the input is identical to the prototype. The prototypes can be selected in different ways. One possibility is to use one prototype for each exemplar that has been encountered. Alternatively, the prototypes can be selected by trying to cover the input space by suitably spaced prototypes. Finally, the prototypes can be found by learning. Once selected, each unit in the middle layer contributes to the output depending on how well the input matches its prototype. RBF-networks have been used in numerous applications for both categorization and function approximation tasks and can easily learn complex relations between their input and output.

A type of RBF-network that is of special interest is normalizing radial basis function networks (Bugmann, 1998). This model differs from the standard model in that the output is normalized. This is an operation that has been suggested to be implemented by lateral inhibition and it is ubiquitous in the brain (Carandini and Heeger, 2012). The importance of the normalization stage is that it makes the output of the RBF-network consist of a convex combination of the outputs of the individual units, where each output is weighed by how close its prototype is to the input. This property guarantees that the output will be a continuous function of the input that quickly converges on the correct mapping during learning. Like other multilayer feed-forward networks, RBF-networks are universal approximators, which means that they can learn any mapping between finite dimensional spaces with any desired accuracy as long as there are a sufficient number of hidden units.

Salinas and Abbott (1995) investigated how the number of units influenced the accuracy of the coding and decoding of different magnitudes. More recently, Eliasmith and Anderson (2003) presented general mathematical recipes for how low dimensional quantities can be coded and decoded in the brain using population codes as well as suggestions for how such quantities can be combined in different ways to implement different arithmetic operations and mappings between spaces. The same type of reasoning can be applied to many different tasks including other sensorimotor transformations, learning and short-term memory (Pouget and Snyder, 2000). This shows that population codes are a general way to code quantities in one- or multi-dimensional spaces and to perform arbitrary operations on them. This further supports that the machinery available to the brain is ideally suited for processing spatial representations.

It is interesting to note that the basis unit coding has many similarities to approaches in control theory, such as locally weighted learning (Atkeson et al., 1997). In fact, Stulp and Sigaud (2015) have shown that many models and algorithms working according to these principles use exactly the same underlying model as the three-layer network described above. This lends support to the idea that there is something fundamental about these types of mechanisms where functions are computed using units that each react to different parts of the input space and the output is subsequently calculated as a combination of the outputs from those individual units.

To summarize, we have proposed that mappings between spaces consist of two steps. The first is a comparison between the input and a number of prototypes and the second is the weighting of the output from each prototype unit by its similarity to the input. The chorus transform provides a good model for the usefulness of population codes, both as a way to represent points in psychological spaces and as a mechanism for mapping between such spaces. RBF-networks constitute the canonical way to model learning of such mappings, but many other models are possible.

## 7. Conclusion

This article has treated two levels of spaces in the brain—psychological and neurocognitive. The psychological spaces, for example the color space, can be studied in psychophysical experiments, in particular with the aid of discrimination or similarity judgments. These spaces can often be represented in a small number of dimensions and we have shown how conceptual spaces can be used to model categorization processes. Neurocognitive representations are implemented implicitly using populations coding where different neurons process different regions of the spaces and allow for efficient mappings between spaces. Furthermore, spatial coding naturally supports generalization from learned examples by interpolation and extrapolation. We have also argued that the psychological spaces naturally emerge from the neural codings.

Although there exist examples of topographic representations in the brain, the spatial representations are typically not topographically organized. This is not even the case for the representations of physical space in the hippocampus. Instead, a population code is used to implicitly represent the spaces. The main advantage of this is that it allows the brain to potentially learn any functional mapping and not only those that can be represented by mappings between two-dimensional spaces.

The main function of spatial representations is to make the mapping from perception to action more efficient. Many models of computations with population codes use explicit representations of perceptual and motor variables. This is useful when investigating what the model is doing, but does not mean that we should expect to find such neurons in the brain, where there is no need to decode the population codes until the final stage when they are used to produce movements. To reveal the low-dimensional spatial representations and to make them match the psychological results, it is necessary to decode the population codes in a low-dimensional space but such a decoding is never explicitly required by the brain itself.

By analyzing the neural representations and reducing them to low-dimensional representations, we have argued that they to a large extent can explain the structure of the psychological spaces. Moreover, we have shown how spatial representations are useful as a basis for categorization and sensory-motor mappings and how they can be implicitly coded by populations of neurons. This suggests that spatial representations can be found everywhere in the brain.

## Author contributions

CB and PG contributed equally to the reported research and the writing of the article.

### Conflict of interest statement

The authors declare that the research was conducted in the absence of any commercial or financial relationships that could be construed as a potential conflict of interest.

## Footnotes

^1^Basis functions are elementary function that can be linearly combined to produce more complex functions in a particular function space.

^2^Euler angles are generally problematic as orientation representations, in particular because they do not fulfill the three criteria of monotonicity, continuity and convexity. However, there exist other representations, such as quarternions, that do.
